# Tribological Performance of Femtosecond Laser-Fabricated Biomimetic Sinusoidal-Circular Composite Textures on 40Cr Steel Under Oil Lubrication

**DOI:** 10.3390/ma19091687

**Published:** 2026-04-22

**Authors:** Yu Chen, Ping Xu, Linhao Zhao, Yinghua Yu, Zipeng Wu

**Affiliations:** 1 School of Mechanical Engineering, Liaoning Technical University, Fuxin 123032, China; 052081@chu.edu.cn (Y.C.); zhusq@chu.edu.cn (Y.Y.); 2 School of Mechanical Engineering, Chaohu University, Hefei 238024, China; 13665613781@163.com (L.Z.);

**Keywords:** 40Cr steel, bionic composite micro-texture, femtosecond laser, coefficient of friction, wear behavior, oil lubrication

## Abstract

To improve the tribological performance of 40Cr steel, a biomimetic composite micro-texture consisting of sinusoidal grooves and circular dimples was designed based on the periodic corrugated structures on the shell surface of *Fimbria fimbriata*. The texture parameter ranges were determined through microscopic characterization of the shell surface and orthogonal design. The composite micro-textures were fabricated on 40Cr steel by femtosecond laser processing and characterized by confocal microscopy, white light interferometry (WLI), and scanning electron microscopy (SEM). Their tribological behavior was evaluated under oil-lubricated reciprocating sliding conditions against a GCr15 counter-body in a ball-on-flat contact configuration. The results showed that laser power significantly affected the forming quality of the sinusoidal textures, and 4.50 W provided the best overall cross-sectional morphology. All textured specimens exhibited lower steady-state average coefficients of friction (COF) than the untextured specimen, with the textured groups ranging from 0.1678 to 0.1905. Among them, specimen L6 showed the lowest steady-state average COF of 0.1678, corresponding to a reduction of approximately 19.4%, together with the best wear resistance as indicated by the relative displacement volume ratio (*K_w_*). Surface analyses revealed that abrasive wear and adhesive wear were the dominant wear mechanisms, while the optimized composite micro-texture effectively suppressed wear-groove development, material pile-up, and transfer-layer formation. Overall, the biomimetic sinusoidal-circular composite micro-texture effectively improved the tribological performance of 40Cr steel under oil lubrication through the synergistic effects of contact-state regulation, lubricant retention, and wear-debris capture. This study provides theoretical and experimental support for the engineering application of biomimetic composite micro-textures on mechanical surfaces.

## 1. Introduction

As mechanical systems continue to advance toward higher performance, longer service life, and greater reliability, friction and wear remain critical challenges in engineering applications. These phenomena directly affect energy efficiency, operational stability, and the durability of key components. In sliding tribological pairs, the contact interface is the core region governing tribological behavior, and its surface topography plays a decisive role in lubrication conditions, oil film stability, and wear mechanisms [1,2,3,4,5]. Therefore, tailoring metallic surface micro-geometries to improve friction reduction and lubrication performance has become an important research focus in surface engineering and tribology.

In recent years, biomimetic micro-texturing inspired by natural surface architectures has attracted increasing attention. Studies have shown that the surfaces of many biological species, including mollusks, fish, and insects, possess micro- and nano-scale structural features evolved through long-term natural selection. These structures often exhibit remarkable functions, such as fluid regulation, drag reduction, anti-adhesion, and wear resistance, and thus provide valuable inspiration for engineering surface design [6,7,8,9,10,11,12,13]. In particular, mollusk shells commonly display regularly distributed corrugated, granular, or composite microstructures. The geometric parameters, periodicity, and spatial arrangement of these features exhibit characteristic patterns, offering useful guidance for the design of functional micro-textures on mechanical surfaces.

Among various micro-texturing methods, femtosecond laser processing has become an effective technique for fabricating high-precision microstructures because of its ultrashort pulse duration, high peak power, and minimal thermal damage [14,15,16,17]. Owing to its non-thermal ablation mechanism, femtosecond laser processing can effectively avoid the large heat-affected zones and thick recast layers typically associated with conventional laser machining. As a result, it is particularly suitable for the precise fabrication of micro-textures on hard metallic materials such as 40Cr steel [18,19]. In addition, by adjusting parameters such as laser power, scanning speed, and scanning strategy, microstructures with controllable depth, width, and periodicity can be produced to satisfy different functional requirements [20,21].

Based on these advantages, increasing attention has been given to combining characteristic biological geometries with specific engineering demands to develop composite micro-textures with enhanced tribological performance. Previous studies have shown that single-type textures, such as grooves, dimples, or linear patterns, can improve tribological behavior under certain operating conditions, but their functional regulation is often limited. By contrast, composite micro-textures integrating different geometric features can simultaneously influence lubricant flow behavior, local contact stress distribution, surface wettability, and wear-debris transport, and may therefore provide more effective and stable tribological regulation than single-type textures [22,23,24,25]. However, most existing studies have focused mainly on single-type textures, while the synergistic tribological regulation mechanism of composite textures remains insufficiently understood. In particular, the coupling relationship between geometric parameter matching and tribological performance under oil lubrication has not yet been systematically clarified for 40Cr steel [26,27,28,29].

As a widely used engineering material for shafts, gears, and other load-bearing components, 40Cr steel possesses good strength, toughness, and wear resistance, and is therefore of considerable practical significance in tribological applications [30,31,32]. Accordingly, the novelty of the present work lies in combining a sinusoidal groove unit for interface regulation and lubricant guidance with a circular micro-pit unit for localized oil storage and wear-debris trapping, thereby establishing a biomimetic composite texture with both biological inspiration and engineering functionality. Therefore, this study designed and fabricated biomimetic sinusoidal-circular composite micro-textures on 40Cr steel by femtosecond laser processing and systematically investigated the relationship between texture parameter combination, tribological behavior, and wear mechanism under oil-lubricated conditions.

## 2. Bionic Composite Micro-Texture Design

### 2.1. Extraction of the Biomimetic Prototype Structure

**Figure 1:**
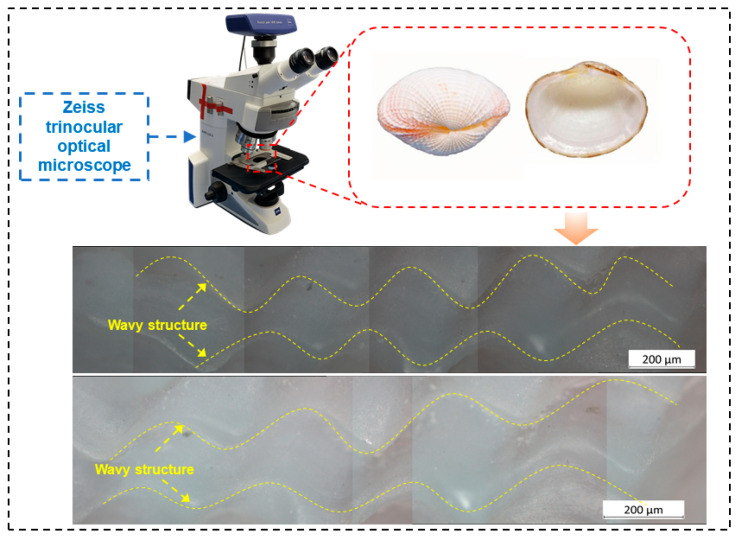
Optical observation of shell surface morphology and periodic wavy structures.

To develop biomimetic micro-textures with both structural similarity and engineering feasibility, the surface morphology of the *Fimbria fimbriata* shell was selected as the biological prototype. Owing to long-term natural evolution, the shell surface has formed microscale corrugated structures with clear periodic features, providing useful inspiration for the design of engineering micro-textures with potential advantages in contact regulation, fluid transport, and wear resistance. The surface morphology of the *Fimbria fimbriata* shell was characterized using a Zeiss trinocular optical microscope, as shown in Figure 1. Four representative regions on the shell surface were selected for observation and parameter extraction. Microscopic images from these regions were collected and stitched to obtain a more complete description of the original surface topography. The results show that the shell surface contains continuous corrugated features distributed along specific directions. The overall morphology is characterized by periodic undulations with alternating peaks and valleys, and similar features were observed in different sampled regions, indicating good morphological consistency.

High-magnification observations further show that the corrugated structures exhibit distinct directional alignment. Because the transitions between peaks and valleys are smooth and continuous, the overall profile can be approximated as a sinusoidal function. Therefore, the natural shell corrugation was simplified as a sinusoidal trajectory for the parametric design of the biomimetic micro-texture. This simplification retains the main topographical characteristics of the biological surface and facilitates the subsequent planning of femtosecond laser scanning paths and processing parameters. Accordingly, the natural shell corrugation was simplified into a sinusoidal groove for biomimetic design. However, considering the limited lubricant retention and wear-debris trapping capacity of single groove textures, circular dimples were further incorporated into the sinusoidal grooves. As a result, a composite micro-texture combining sinusoidal grooves and circular dimples was designed to improve the overall tribological performance of the surface.

### 2.2. Measurement of Corrugation Parameters

**Figure 2:**
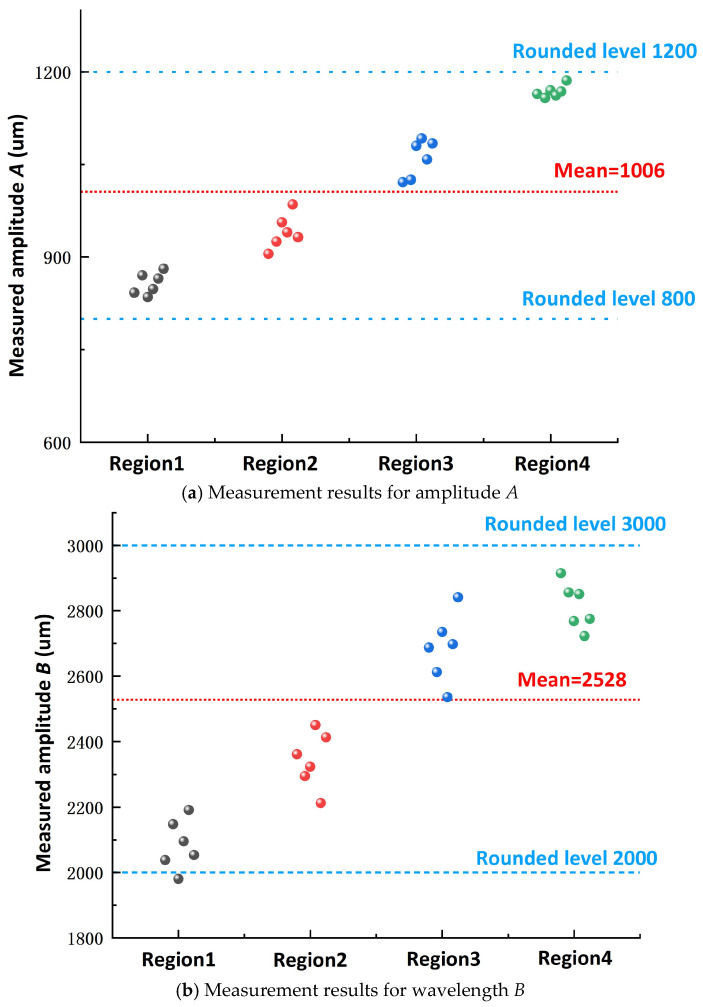

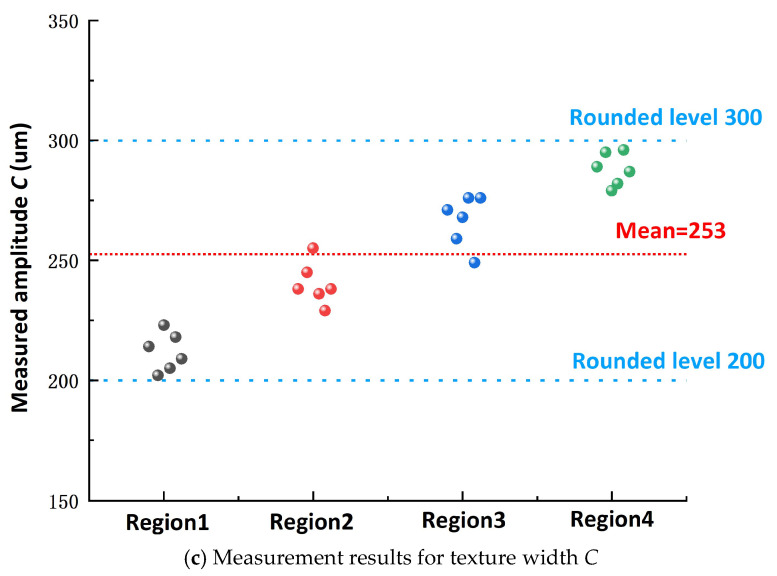
Statistical distributions of the measured corrugation parameters across different regions of the Fimbria fimbriata shell. Black, red, blue, and green dots represent the measured data from Region 1, Region 2, Region 3, and Region 4, respectively.

To improve the reliability of biomimetic parameter extraction, the geometric parameters of the corrugated structures on the Fimbria fimbriata shell surface were measured based on images acquired using a Zeiss trinocular optical microscope. As shown in Figure 2, three main geometric parameters were defined in representative corrugated regions, including amplitude (*A*), wavelength (*B*), and texture width (*C*). Amplitude (*A*) was used to describe the height variation of the corrugated profile, wavelength (*B*) represented the spatial period of the profile, and texture width (*C*) denoted the lateral width of a single corrugated band.

To avoid the uncertainty associated with single-region measurements, four representative regions of the *Fimbria fimbriata* shell were selected for parameter measurement. Multiple corrugated units in each region were repeatedly measured to obtain raw data for *A*, *B*, and *C*, and the results were then statistically analyzed. During measurement, the amplitude (*A*) was determined as the vertical distance between the peak and valley of a representative corrugation unit, the wavelength (*B*) was defined as the horizontal distance between two adjacent peaks, and the texture width (*C*) was taken as the lateral width of a single corrugated band. This multi-region and repeated-measurement strategy provided a representative description of the shell surface morphology and formed the basis for subsequent parameter normalization and orthogonal factor design.

### 2.3. Statistical Analysis and Range Determination of Biomimetic Parameters

Based on the optical measurement data obtained from four representative regions, statistical analysis was performed on the amplitude (*A*), wavelength (*B*), and texture width (*C*) of the Fimbria fimbriata shell surface. The corresponding statistical distributions and results are shown in Figure 2a–c.

As shown in Figure 2a, although the measured amplitude *A* values from different regions exhibit some variation, their overall distribution is relatively concentrated, mainly within the range of approximately 900–1170 μm, with an average value of about 1006 μm. Accordingly, three levels were selected for the amplitude parameter: 800 μm, 1000 μm, and 1200 μm.

As shown in Figure 2b, the measured wavelength *B* values are mainly distributed between 2200 and 2820 μm, with an average value of approximately 2450 μm. By considering biomimetic similarity, manufacturing feasibility, and the need for parameter discretization, parameter B was normalized into three levels: 2000 μm, 2500 μm, and 3000 μm.

As shown in Figure 2c, the measured texture width *C* ranges from 230 to 305 μm, with an average value of approximately 280 μm. To ensure profile integrity, boundary clarity, and sufficient distinction among parameter combinations during subsequent femtosecond laser fabrication, parameter *C* was set at three levels: 200 μm, 250 μm, and 300 μm.

Based on microscopic characterization, multi-region measurement, and statistical normalization, the parameter ranges of the biomimetic sinusoidal texture were determined for subsequent orthogonal design. The selected factor levels retained the main geometric scale of the biological prototype while ensuring sufficient distinction and manufacturability in femtosecond laser fabrication.

### 2.4. Determination of Composite Texture Parameters and Orthogonal Design

Building upon the natural corrugated architecture of the shell surface, circular dimples were incorporated into the sinusoidal textures to construct a biomimetic composite micro-texture. In this composite system, the sinusoidal grooves mainly function to regulate the contact interface morphology during sliding and to facilitate continuous lubricant transport, while the circular dimples provide localized lubricant storage and wear-debris trapping. Through the combination of these two structural features, the tribological performance of the surface is expected to be enhanced.

Because the circular dimples were not directly derived from the biological prototype of the *Fimbria fimbriata* shell, their diameter (*D*) was not determined from optical measurements. Instead, parameter *D* was established by considering the requirements of composite functional synergy, surface load-carrying capacity, and the processing accuracy of femtosecond laser fabrication. To balance lubricant retention and debris capture with manufacturing feasibility, the diameter of the circular dimples was set at three levels: 40 μm, 60 μm, and 80 μm.

To ensure fabrication consistency and improve comparability among different specimens, the depths of the sinusoidal grooves (*E*) and circular dimples (*F*) were kept constant at 100 μm and 50 μm, respectively. The laser scanning speed and scanning pitch were also maintained constant. Therefore, the orthogonal design focused on four geometric factors only: sinusoidal amplitude (*A*), sinusoidal wavelength (*B*), texture width (*C*), and circular dimple diameter (*D*). Based on the statistical results shown in Figure 2 and the design requirements of the composite micro-texture, the factors and levels for the orthogonal experiment were determined, as listed in Table 1. This treatment reduced the number of variables in the orthogonal design and improved the comparability among different parameter combinations, allowing the analysis to focus on the influence of the major in-plane geometric factors of the composite texture.

As listed in Table 1, the orthogonal design focused on four main geometric factors, namely sinusoidal amplitude (*A*), sinusoidal wavelength (*B*), texture width (*C*), and circular dimple diameter (*D*). The selected levels were determined by combining the statistical characteristics of the biological prototype with the manufacturability requirements of femtosecond laser fabrication. This design enabled the systematic investigation of the influence of different parameter combinations on the tribological performance of the biomimetic composite micro-texture.

After the factor levels were determined, a standard Taguchi L9 orthogonal array was adopted to generate nine parameter combinations for the biomimetic composite micro-textured specimens, as listed in Table 2. This design allowed the effects of multiple factors and levels to be evaluated systematically while reducing the total number of experimental runs. The nine groups of specimens prepared according to this orthogonal scheme were subsequently used for femtosecond laser fabrication, surface characterization, and tribological testing, thereby enabling analysis of the relationships between texture parameter combination, forming quality, friction coefficient, and wear behavior.

## 3. Specimen Preparation and Experimental Methods

### 3.1. Specimen Material and Pretreatment

In this study, 40Cr steel in the hot-rolled condition was used as the substrate material for the fabrication of composite micro-textured specimens. The specimens were machined into blocks with dimensions of 20 mm × 20 mm × 5 mm. The hot-rolled substrate was selected because its relatively uniform microstructural state and mechanical properties helped reduce the influence of material heterogeneity on the tribological test results. Before laser texturing, all specimens were uniformly ground with metallographic sandpapers of different grit sizes to remove machining marks and then polished to obtain a smooth and consistent initial surface condition. After polishing, the specimens were ultrasonically cleaned in acetone, anhydrous ethanol, and deionized water in sequence to remove residual oil, particles, and other contaminants, and were finally air-dried for subsequent processing. The initial surface roughness after pretreatment was approximately Ra < 0.1 μm, indicating that a smooth and uniform surface condition was achieved before laser texturing.

### 3.2. Fabrication of Composite Micro-Textured Specimens

Following the biomimetic parameter extraction and orthogonal design presented in Section 2, sinusoidal-circular composite micro-textures were fabricated on 40Cr steel surfaces using femtosecond laser processing. Owing to its ultrashort pulse duration, minimal heat-affected zone, and high machining precision, femtosecond laser processing is well suited for the fabrication of complex microscale textures. This method therefore meets the requirements for the high-precision preparation of the bionic composite micro-textures used in this study.

**Figure 3:**
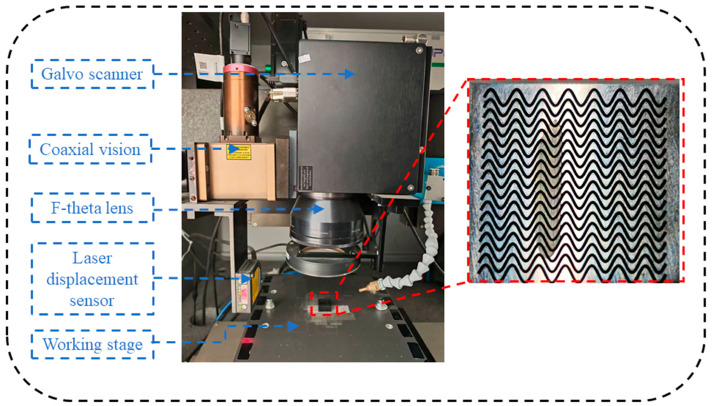
System configuration and fabrication results. The QR code visible on the equipment is part of the original device image and is not related to the experimental results.

The femtosecond laser processing system and the fabricated composite micro-textures are shown in Figure 3. The system mainly consists of a galvanometer scanning system, a coaxial vision system, an F-theta objective lens, a laser displacement sensor, and a high-precision translation stage. During processing, the 40Cr specimen was fixed on the stage, and the laser beam was deflected by the galvanometer and focused onto the specimen surface through the F-theta lens. By scanning along the preset paths, the designed micro-textures were fabricated on the 40Cr surface. The laser displacement sensor was used to maintain the focal position and ensure processing stability, while the coaxial vision system enabled accurate positioning and real-time monitoring of the processing area.

The composite micro-texture consisted of continuous sinusoidal grooves combined with circular dimples. During fabrication, the sinusoidal grooves were first generated along the designed paths, and the circular dimples were then introduced at specific locations. As shown in Figure 3, the femtosecond laser processing system produced regular composite texture arrays with clear profiles on the 40Cr specimens, indicating that the system was suitable for the fabrication of the designed bionic composite micro-textures.

During specimen preparation, the geometric parameters, including amplitude (*A*), wavelength (*B*), texture width (*C*), and circular dimple diameter (*D*), were varied according to the L9 orthogonal design established in Section 2. To ensure comparability among different specimens, the depths of the sinusoidal grooves (*E*) and circular dimples (*F*) were kept constant at 100 μm and 50 μm, respectively. In addition, the laser scanning speed and hatch spacing were also maintained constant. In this way, the performance differences among specimens could be mainly attributed to the texture geometry rather than variations in processing conditions.

Based on the orthogonal design, nine composite micro-textured specimens, denoted as L1–L9, were fabricated. These specimens were used in the subsequent analyses of surface morphology, reciprocating friction and wear behavior, and wear volume, so as to clarify the relationship between texture parameter combinations and tribological performance.

### 3.3. Laser Processing Parameter Optimization and Surface Characterization

Before batch fabrication, the femtosecond laser processing parameters were optimized to obtain composite micro-textures with clear profiles and stable dimensions. Based on preliminary single-factor experiments, this study mainly investigated the effect of laser power on the cross-sectional morphology of the sinusoidal textures. The optimized results were then used to determine the processing parameters for the composite micro-textured specimens. The experimental conditions for laser power optimization are listed in Table 3.

During the power optimization process, all parameters except laser power were kept constant, including a scanning speed of 400 mm/s, a repetition rate of 100 kHz, a scanning pitch of 5 μm, and 75 scanning passes. Under these conditions, the differences in texture profile could be mainly attributed to the variation in laser power.

**Figure 4:**
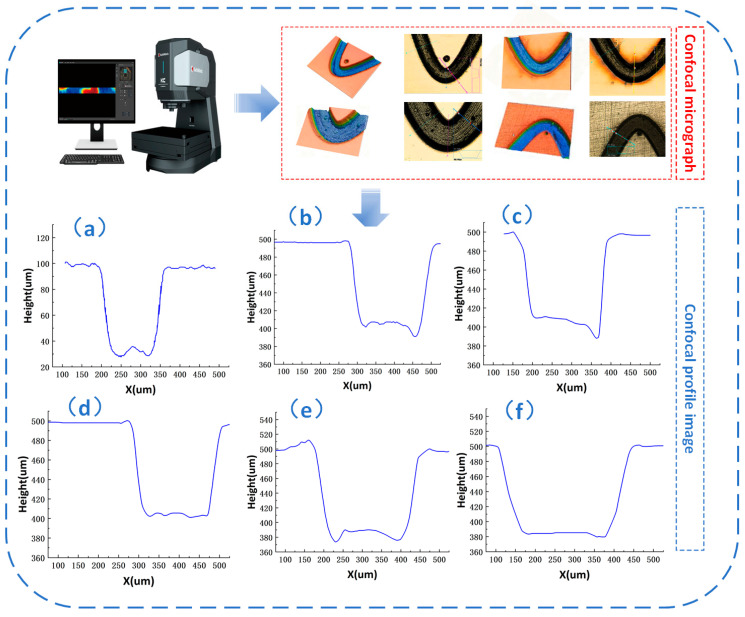
Confocal morphologies and cross-sectional profiles of sinusoidal textures fabricated under different laser powers. Panels (**a**–**f**) correspond to laser powers of 3.75, 4.00, 4.25, 4.50, 4.75, and 5.00 W, respectively. The nearly horizontal regions on both sides of each profile represent the untreated reference surface, whereas the central concave region indicates the laser-fabricated textured groove. The blue arrow is a schematic indicator and is used only to guide the correspondence between the confocal image and the extracted profile.

The confocal morphologies and corresponding cross-sectional profiles of the sinusoidal textures processed at different laser powers are shown in Figure 4. Panels (a)–(f) correspond to laser powers of 3.75, 4.00, 4.25, 4.50, 4.75, and 5.00 W, respectively. As shown in Figure 4, laser power has a significant effect on the forming quality of the textures.

At lower laser powers, insufficient material removal led to shallow grooves and unclear boundaries, which did not meet the design requirements. As the laser power increased, the groove depth gradually increased, and the cross-sectional profiles became more complete, with improved sidewall quality. However, at higher laser powers, although the material removal capacity was further enhanced, local defects such as increased bottom fluctuations and irregular edges began to appear. This indicates that excessively high laser power may reduce processing stability and deteriorate the geometric accuracy of the textures.

A comparison of the confocal morphologies, groove depths, and cross-sectional profiles at different laser powers indicates that 4.50 W produced textures closest to the design requirements. At this power, the groove boundaries were clear, the cross-sectional profile was complete, and no obvious over-ablation or geometric distortion was observed. Therefore, 4.50 W was selected as the optimal laser power for fabricating the sinusoidal textures. At a repetition rate of 100 kHz, this corresponds to a single-pulse energy of approximately 45 μJ.

For the circular dimples, the processing parameters were determined based on preliminary experiments. A single-pulse energy of 16 μJ, a repetition rate of 10 kHz, a scanning speed of 10 mm/s, a scanning pitch of 5 μm, and 12 scanning passes were adopted. Under these conditions, corresponding to an average laser power of approximately 0.16 W, circular dimples with a depth of about 50 μm could be stably fabricated. This parameter set provided good dimensional consistency and facilitated the integration of the dimples with the sinusoidal grooves to form the composite micro-texture.

As shown in Figure 4, under the optimized conditions, femtosecond laser processing produced textures with clear contours and stable depths on the 40Cr surface. In particular, the depth of the sinusoidal grooves was close to the target value of 100 μm, and the cross-sectional profile showed good continuity. Combined with the processing parameters listed in Table 3, these results indicate that the optimized process can stably fabricate bionic textures with good geometric consistency, thereby providing a reliable basis for subsequent composite micro-texture evaluation and reciprocating tribological tests.

### 3.4. Tribological Testing and Wear Characterization

**Figure 5:**
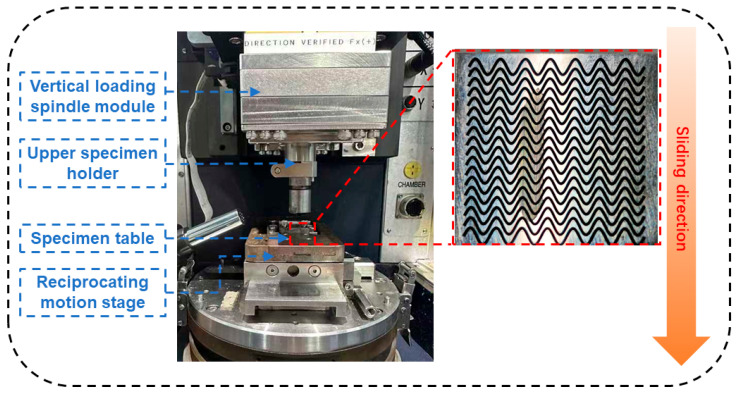
Schematic of the reciprocating friction and wear test setup (adapted from Ref. [33]).

To evaluate the effect of the biomimetic sinusoidal-circular composite micro-textures on the tribological performance of 40Cr steel, reciprocating friction and wear tests were carried out using a Bruker CETR UMT-2 tribometer. The test setup and contact configuration are shown in Figure 5. During the tests, the composite micro-textured 40Cr specimens served as the lower samples and were fixed on the test platform, while a GCr15 steel ball with a diameter of 6 mm was used as the counter-body material. All tests were performed under oil-lubricated conditions using No. 68 guideway oil.

The test parameters are listed in Table 4. A constant normal load of 150 N was applied, with a reciprocating stroke of 10 mm and a frequency of 5.0 Hz, corresponding to an average sliding speed of 100 mm/s. Under these conditions, the tribological pair underwent periodic reciprocating motion, and the coefficient of friction (COF) was recorded in real time during the tests to evaluate the friction reduction performance of specimens with different texture parameter combinations. It should be noted that the present reciprocating ball-on-flat tribological test was intended as a comparative screening condition for evaluating the relative tribological performance of different texture parameter combinations under controlled oil-lubricated conditions, rather than a direct simulation of the actual contact state of shafts or gears in service.

The friction coefficient curves generally consisted of an initial running-in stage followed by a relatively stable stage. During the running-in stage, the friction coefficient showed relatively large fluctuations because of the contact adaptation between surface asperities and the redistribution of the lubricant film. As sliding continued, the contact interface gradually stabilized, and the friction coefficient entered a steady-state stage. Therefore, the tribological performance of different composite micro-textured specimens was evaluated mainly based on the full friction curves and the average COF in the steady-state stage. Single tribological tests were performed for each specimen under the same test conditions, and the COF data were processed and compared using a unified procedure.

After the friction tests, the worn regions were characterized by white light interferometry (WLI) to further evaluate wear resistance. WLI provides high-precision surface topography data and enables three-dimensional reconstruction of the wear scars. Based on the WLI results, the wear scar width, depth, and surface morphology could be quantitatively analyzed. To compare the wear behavior of different specimens, the unworn surface adjacent to the wear scar was used as the reference region, and the corresponding topographic data were further processed to evaluate the relative material displacement in the worn area.

For subsequent wear evaluation, the volumetric parameters obtained from WLI were defined as follows. *V_n_* denotes the natural volume of the analyzed wear-scar region defined by the reconstructed reference topographic envelope, and *V_d_* denotes the total displaced volume within the same analyzed region. Their ratio, *K_w_* = *V_d_*/*V_n_*, was used as a normalized wear indicator to compare the relative degree of material displacement among different specimens. A lower *K_w_* value indicates lower relative material displacement and thus comparatively better wear resistance under the present test conditions.

In addition, scanning electron microscopy (SEM) was used to observe the surface morphology before and after the friction tests in order to analyze the wear characteristics. By comparing the original textured surface with the worn morphology after testing, typical features such as abrasive grooves, local plastic deformation, material transfer, and debris accumulation could be identified. These observations were used to interpret the dominant wear characteristics of the textured surfaces. By combining the COF curves, WLI results, and microscopic morphology observations, the friction reduction and antiwear mechanisms of the biomimetic composite micro-textures were systematically analyzed.

## 4. Results and Discussion

### 4.1. Influence of Laser Power on Sinusoidal Texture Cross-Sectional Morphology

**Figure 6:**
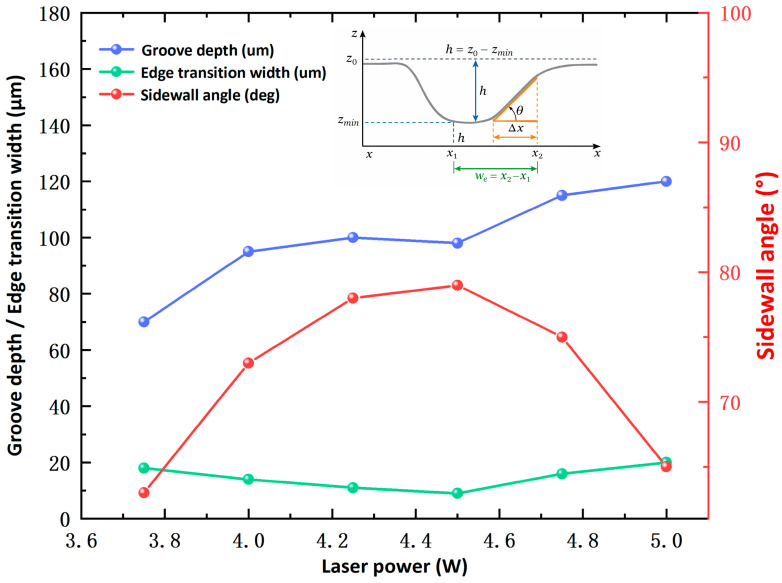
Confocal microscopic morphologies and cross-sectional profiles of sinusoidal textures at various laser powers.

To obtain sinusoidal textures with good forming quality, the cross-sectional morphologies produced at different laser powers were compared before the systematic fabrication of the composite micro-textured specimens. The confocal morphologies and corresponding cross-sectional profiles at different laser powers have been shown in Figure 4, where panels (a)–(f) correspond to laser powers of 3.75, 4.00, 4.25, 4.50, 4.75, and 5.00 W, respectively. To further evaluate the fabrication quality quantitatively, several geometric parameters, including groove depth, sidewall angle, and edge transition width, were extracted from the cross-sectional profiles. It should be noted that the nearly horizontal regions on both sides of the measured profile correspond to the untreated reference surface, whereas the central depressed region represents the laser-textured groove. Therefore, the measured cross-sectional profile inherently contains both the pre-texturing reference level and the post-texturing morphology, allowing direct evaluation of the surface-topography change induced by laser processing. Their definitions and variation trends are shown in Figure 6, and the corresponding quantitative results are listed in Table 5. The geometric parameters shown in Figure 6 and Table 5 were calculated directly from representative measured profile curves under each laser power condition and were used for comparative evaluation of the texture-forming quality.

The groove depth (*h*) is defined as the vertical distance between the reference height of the unprocessed surface (*z*_0_) and the minimum height of the groove (*z_min_*):

(1) $$h=z_{0}-z_{min}$$

The sidewall angle (*θ*) characterizes the steepness of the groove walls. It is calculated based on the height difference and the horizontal distance within the approximately linear region of the sidewall:

(2) $$\theta =arctan(\frac{∆h}{∆x})$$

The edge transition width (*w_e_*) represents the horizontal extent of the transition zone from the unprocessed surface to the groove sidewall. A smaller we value signifies a more concentrated texture boundary and a sharper edge:

(3) $$w_{e}=x_{2}-x_{1}$$

As shown in Figure 4 laser power has a significant effect on the cross-sectional morphology of the sinusoidal textures. In general, increasing the laser power enhanced the material removal capability, causing the groove profile to change from shallow and unclear to deep and well defined. At low laser powers, insufficient energy input led to incomplete material removal, resulting in shallow grooves with blurred boundaries that could not meet the target depth. As the power increased, the groove profile became more complete, the sidewalls became clearer, and the boundary definition improved.

Table 5 summarizes the geometric parameters extracted from the sinusoidal texture cross-sections at different laser powers. As the power increased from 3.75 W to 5.00 W, the groove depth increased from 70 μm to 120 μm, indicating that higher laser power enhanced the material removal rate. In particular, at 4.25 W and 4.50 W, the measured groove depths were 100 μm and 98 μm, respectively, which were closest to the target value of 100 μm. However, when the power was further increased to 4.75 W and 5.00 W, the groove depths increased to 115 μm and 120 μm, respectively, exceeding the design value. This suggests that excessive laser power improves removal efficiency but reduces dimensional accuracy.

For the sidewall angle (*θ*), the value increased from 63° to 79° as the power increased from 3.75 W to 4.50 W, indicating that the sidewalls became steeper and the boundary transition became clearer. This suggests that a moderate increase in laser power improved sidewall forming quality. However, when the power was further increased to 4.75 W and 5.00 W, the sidewall angle decreased to 75° and 65°, respectively. This reduction may be attributed to stronger localized melting and resolidification at excessive power, which caused boundary blunting and reduced profile regularity.

The variation in edge transition width (*w_e_*) further reflects the boundary sharpness. As listed in Table 5, when the power increased from 3.75 W to 4.50 W, we decreased from 18 μm to 9 μm, indicating a gradual improvement in edge sharpness. However, at 4.75 W and 5.00 W, we increased to 16 μm and 20 μm, respectively, suggesting that excessive energy input led to boundary broadening. Therefore, 4.50 W produced the minimum we value and the best edge quality.

The trends shown in Figure 6 indicate that laser power effectively regulates the forming quality of sinusoidal textures. Although the groove depth generally increased with laser power, the sidewall angle first increased and then decreased, while the edge transition width first decreased and then increased. These results indicate that there is an optimal laser power range for texture fabrication. Excessively increasing the power does not further improve the texture quality and may instead lead to edge blunting and profile distortion. Therefore, in addition to groove depth, parameters such as sidewall angle and edge transition width should also be considered in the evaluation of texture forming quality.

### 4.2. Friction Coefficient Evolution of Different Composite Micro-Textured Specimens

**Figure 7:**
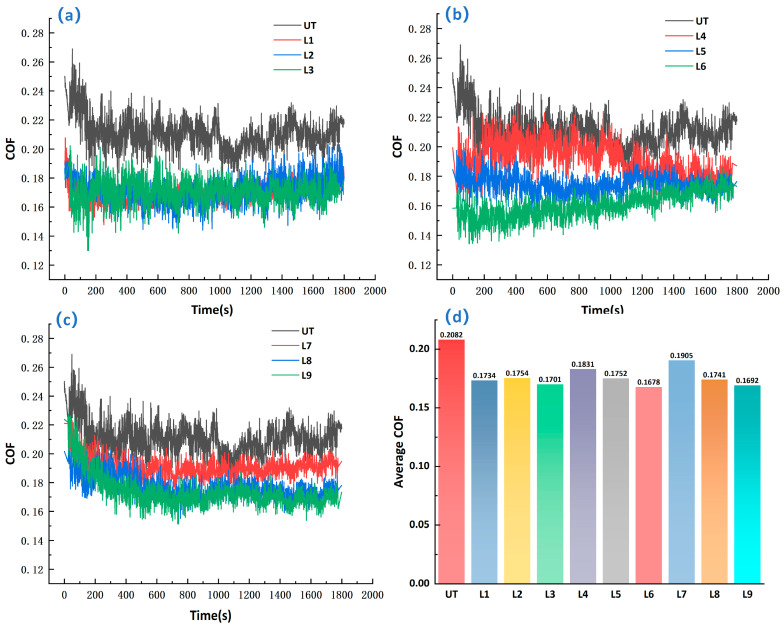
Friction coefficient curves and steady-state average COF values of different specimens: (**a**) UT, L1, L2, and L3; (**b**) UT, L4, L5, and L6; (**c**) UT, L7, L8, and L9; (**d**) steady-state average COF values of UT and L1–L9.

The friction coefficient (COF) curves and the corresponding steady-state average COF values of different specimens under oil-lubricated conditions are shown in Figure 7a–d. Figure 7a–c present the time-dependent COF curves of the different textured groups in comparison with the untextured specimen, while Figure 7d summarizes the steady-state average COF values of UT and L1–L9. To reduce the influence of the initial running-in stage, the COF data from the last 40% of the test duration were defined as the steady-state stage, and the average value within this interval was used to evaluate the stable friction performance of each specimen.

As shown in Figure 7a–c, the COF curves of all specimens exhibited two typical stages: an initial running-in stage and a steady-state stage. At the beginning of sliding, the COF values were relatively high and fluctuated markedly because of the initial contact adaptation between surface asperities and the unstable redistribution of the lubricant film. As sliding continued, the contact interface gradually stabilized, and the COF of each specimen entered a relatively steady stage with smaller fluctuations.

Overall, all composite micro-textured specimens (L1–L9) showed lower COF values than the untextured specimen (UT), indicating that the biomimetic sinusoidal-circular composite micro-textures improved the frictional behavior of 40Cr steel under oil-lubricated conditions. The UT specimen maintained a relatively high COF throughout the test, with a steady-state average value of 0.2082, which was higher than those of all textured specimens. This suggests that the untextured surface had weaker capacities for lubricant retention, transport, and wear-debris capture, making it difficult to maintain a stable lubrication state.

According to Figure 7d, the steady-state average COF values were 0.1734 for L1, 0.1754 for L2, 0.1701 for L3, 0.1831 for L4, 0.1752 for L5, 0.1678 for L6, 0.1905 for L7, 0.1741 for L8, and 0.1692 for L9. Among them, L6 showed the lowest steady-state average COF of 0.1678, followed by L9 (0.1692) and L3 (0.1701). Although L7 exhibited the highest COF among the textured specimens (0.1905), it still performed better than UT. These results indicate that the friction reduction performance varied noticeably with the texture parameter combination.

Compared with UT, the steady-state average COF values of L6, L9, and L3 decreased by approximately 19.4%, 18.7%, and 18.3%, respectively, indicating favorable friction reduction behavior. In contrast, L7 showed a reduction of only about 8.5%, suggesting that this parameter combination was less effective in improving the lubrication condition and contact state. In general, all textured specimens showed lower friction than the untextured surface.

From the curve characteristics, specimens with better friction reduction performance generally had shorter running-in periods and more stable steady-state behavior. For example, L3, L6, and L9 quickly reached a stable state and showed relatively small fluctuations, indicating that their texture geometries were more favorable for lubricant retention and interface regulation. In contrast, L4 and L7, especially L7, maintained relatively high COF values during the steady-state stage, suggesting that their parameter combinations did not fully realize the synergistic effect of sinusoidal grooves and circular dimples.

To further evaluate the influence of texture parameters on the steady-state friction behavior, range analysis was carried out based on the average COF values of specimens L1–L9 obtained from the last 40% of the test duration, and the results are summarized in Table 6. According to the range values, the influence of the factors on the steady-state average COF followed the order of *B* > *C* > *D* > *A*, indicating that the sinusoidal wavelength had the strongest effect within the investigated range, followed by the texture width and the circular dimple diameter, while the sinusoidal amplitude showed the least effect. Taking the minimum average COF as the optimization objective, the theoretical optimal combination was determined as *A*1 *B*3 *C*1 *D*1. This result further indicates that the tribological performance of the biomimetic composite textures is closely related to the geometric parameter combination.

In summary, the biomimetic sinusoidal-circular composite micro-textures effectively reduced the friction coefficient of 40Cr steel under oil-lubricated conditions, but the performance was strongly affected by the texture parameter combination. Among the tested specimens, L6, L9, and L3 exhibited comparatively lower steady-state average COF values, whereas L7 and L4 showed relatively less favorable friction reduction behavior. The following sections further analyze the anti-wear behavior and related mechanisms based on wear volume and surface morphology results.

### 4.3. Analysis of Wear Behavior and Surface Topography

**Figure 8:**
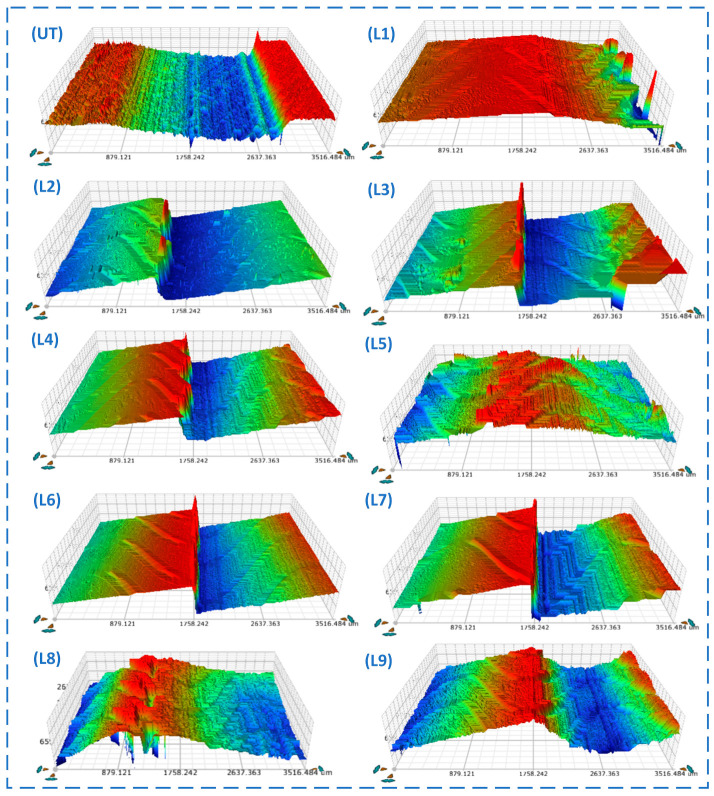
White-light interferometric 3D morphologies of wear scars for different specimens. The color scale represents the surface height distribution, where warmer colors indicate relatively higher regions and cooler colors indicate relatively lower regions. UT denotes the untextured specimen, while L1–L9 denote the textured specimens corresponding to the different orthogonal design groups.

To further evaluate the wear resistance of different composite micro-textured specimens under oil-lubricated conditions, the worn regions were characterized by white light interferometry (WLI), and the three-dimensional surface morphologies are shown in Figure 8. As shown in the figure, the wear scar morphologies showed clear differences among the specimens, indicating that the geometric parameter combinations of the composite micro-textures had an important influence on wear behavior. Overall, the untextured specimen (UT) exhibited a wider and deeper wear scar with more pronounced surface fluctuations, suggesting more severe material removal during sliding. In contrast, most composite micro-textured specimens showed shallower worn regions and weaker local undulations, indicating that the biomimetic composite micro-textures effectively improved the lubrication condition and reduced wear.

To quantitatively evaluate the wear severity of different specimens, the relative displacement volume ratio *K_w_* was calculated based on the WLI-derived volumetric parameters defined in Section 3.4:

(4) $$K_{w}=\frac{V_{d}}{V_{n}}$$

Here, *V_d_* represents the total displaced volume and *V_n_* represents the natural volume of the analyzed region. A lower *K_w_* value indicates lower relative material displacement and better wear resistance.

As indicated in Table 7, the wear severity varies significantly among the different composite micro-textured specimens. The untextured specimen (UT) exhibited a *K_w_* of 0.601, indicating severe overall wear and poor wear resistance. Specimen L1 recorded a *K_w_* of 0.630, slightly exceeding that of UT, which suggests that this specific parameter combination failed to effectively improve surface durability. Conversely, L6 achieved the minimum *K_w_* of 0.255, representing the lowest degree of relative material displacement within the analyzed area and indicating comparatively favorable wear resistance under the present test conditions. Specimens L3 and L2 followed with *K_w_* values of 0.270 and 0.316, respectively, also exhibiting relatively mild wear. While L8, L7, L9, and L5 showed moderate wear levels, L4 yielded a *K_w_* of 0.519, reflecting relatively heavy wear damage.

Correlating these findings with the 3D wear scar morphologies in Figure 8 further reveals that specimens with superior wear resistance typically feature shallower wear regions, gentler surface undulations, and minimal localized height variations. For instance, the overall profiles of L6, L3, and L2 remain relatively flat, indicating limited material removal and controlled wear progression during the sliding process. In contrast, UT, L1, and L4 exhibit pronounced height gradients and deeper wear grooves, reflecting severe material migration and interfacial damage. These observations suggest that optimized composite texture parameters can effectively alleviate localized contact stress concentrations and improve the distribution of lubricating oil across the interface, thereby reducing material removal.

The specimens with lower steady-state average COF, especially L6 and L3, generally also exhibited lower relative wear severity and milder post-friction surface damage, indicating that the favorable texture parameter combinations simultaneously improved lubrication stability and suppressed material removal. However, the relationship between COF and wear was not strictly linear, suggesting that interfacial friction and accumulated wear were jointly influenced by lubricant retention, debris trapping, and local material transfer.

A comparison between the wear results and the previously discussed friction coefficient (COF) data demonstrates a high degree of consistency between the friction reduction and wear-resistance performance of the composite micro-textured specimens. L6 not only achieved the lowest average COF but also the minimum *K_w_*, showcasing exceptional comprehensive performance in both metrics. L3 similarly performed well in both categories, indicating that its textural configuration effectively enhances the interfacial lubrication state while inhibiting material removal. Although L9 showed superior friction reduction performance, its *K_w_* was slightly higher than those of L6 and L3, suggesting that while friction and wear are correlated, they do not maintain a strictly linear relationship. Meanwhile, the heavy wear observed in L4 and L1 aligns with the overall trend of their relatively high friction coefficients.

From a mechanistic perspective, the bionic sinusoidal–circular composite micro-textures improve surface wear behavior through multiple synergistic effects. The sinusoidal textures modulate the contact morphology of the friction interface, facilitating the guidance and redistribution of the lubricant within the contact zone. Simultaneously, the circular dimples serve as localized oil reservoirs and traps for wear debris, mitigating the recurrent grinding action of abrasive particles at the interface. When the parameters of these two texture types are optimally matched, a more stable lubrication state is established during sliding, which lowers localized contact stresses and minimizes material removal, resulting in superior wear resistance. Conversely, suboptimal geometric combinations may undermine the capacities for fluid guidance, oil storage, and debris sequestration, ultimately leading to significant material displacement and severe surface damage.

### 4.4. Surface Micro-Morphology and Wear Mechanism Analysis

**Figure 9:**
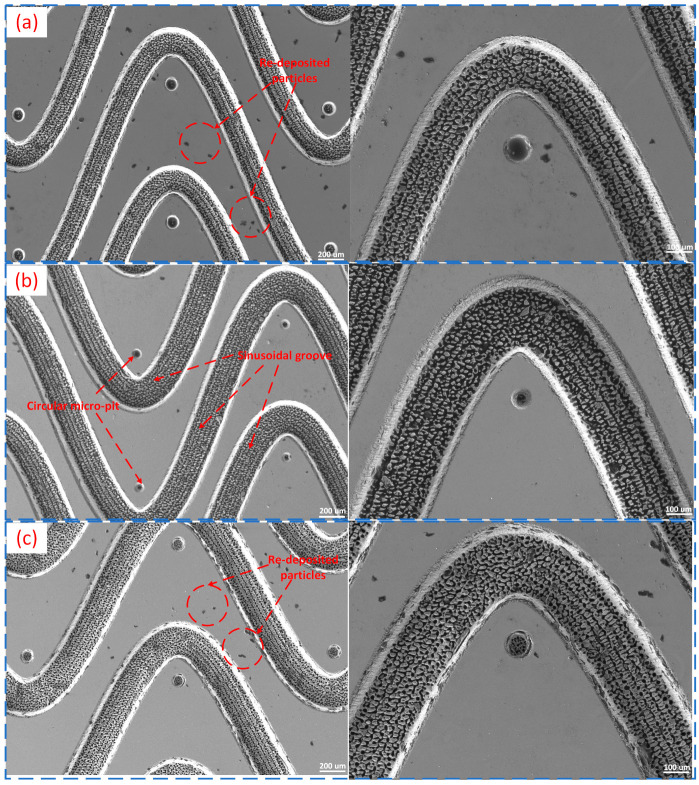
Surface morphologies of the laser-fabricated composite micro-textures before the friction tests: (**a**) specimen L6; (**b**) specimen L7; and (**c**) specimen L8. The sinusoidal grooves and circular micro-pits were successfully fabricated on all three specimens. Localized re-deposited particles can be observed in some regions, which are attributed to melting, splashing, and rapid-cooling redeposition during femtosecond laser ablation. For each specimen, the left and right panels show views at different magnifications.

**Figure 10:**
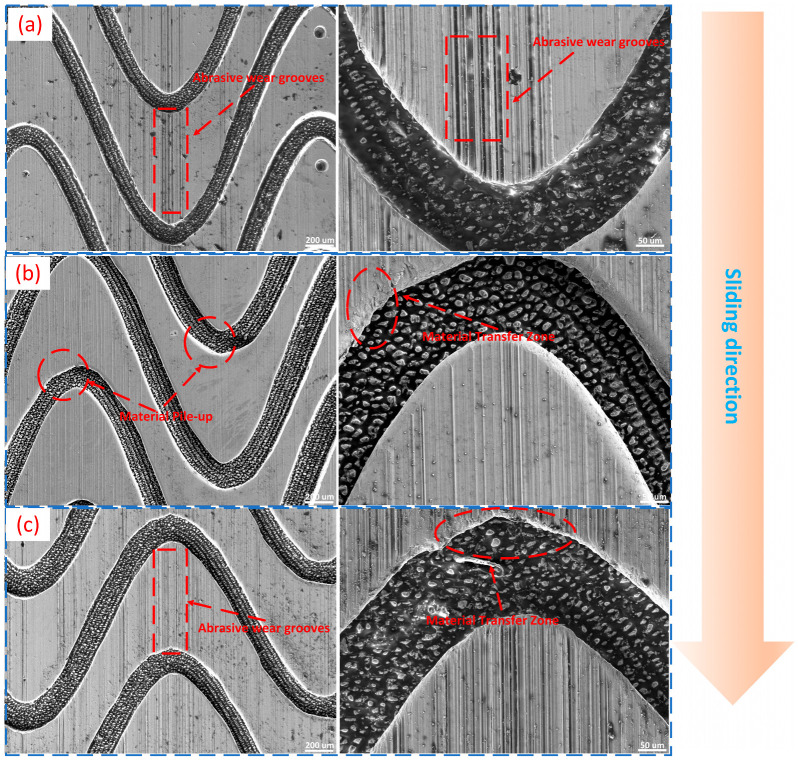
SEM morphologies of the wear scars on representative composite-textured specimens after the friction tests: (**a**) specimen L6; (**b**) specimen L7; and (**c**) specimen L8. For each specimen, the left image shows the overall wear scar, whereas the right image shows a locally enlarged view.

To elucidate the underlying causes of the performance disparities among the various composite micro-textured specimens, scanning electron microscopy (SEM) was employed to characterize the surface micro-morphology of representative specimens (L6, L7, and L8) both before and after the friction tests. The results are presented in Figure 9 and Figure 10.

As shown in Figure 9, well-defined composite micro-textures were successfully fabricated on the surfaces of the three specimens prior to the tests. The continuously distributed sinusoidal grooves and periodically arranged circular micro-pits exhibit complete profiles, indicating that the femtosecond laser processing effectively realized the predefined textural architectures. Minimal re-deposited particles are visible in localized regions, primarily resulting from material melting, splashing, and subsequent rapid-cooling redeposition during the laser ablation process. Overall, L6 possesses sharper texture boundaries and fewer surface-attached particles, representing a better initial surface state than L7 and L8. L7 exhibits more pronounced localized edge undulations and particle attachment, while the surface quality of L8 falls between that of L6 and L7.

The post-test surface morphologies are illustrated in Figure 10. Abrasive wear grooves of varying degrees were observed across the wear scars of all three specimens, indicating that abrasive wear was the dominant wear mode. In addition, localized plastic deformation and material transfer features were observed in some worn regions, suggesting that local material flow and possible adhesive interaction also occurred during the sliding process. Therefore, under oil-lubricated conditions, the wear behavior of these specimens can be interpreted mainly as abrasive wear accompanied by localized material transfer and deformation.

Further comparison of the three specimens reveals that L6 exhibited the lightest surface damage after friction, characterized by shallower wear grooves and weaker localized deformation and material transfer. In contrast, L7 displayed the most pronounced surface impairment, with deeper wear grooves and more evident local deformation and transfer features. The damage severity of L8 was intermediate between that of L6 and L7. These observations are generally consistent with the previously discussed friction coefficient and wear evaluation results, confirming that specimens with superior comprehensive performance typically exhibited milder post-friction surface damage.

It should also be noted that, in the present configuration, the reciprocating sliding direction was arranged along the principal orientation of the sinusoidal texture. This directional relationship was beneficial for lubricant guidance and wear-debris transport along the groove direction and should therefore be considered when interpreting the friction and wear results. The results above indicate that the friction reduction and antiwear efficacy of the composite micro-textures stem primarily from their synergistic regulation of interfacial contact state and lubrication conditions. Specifically, the sinusoidal textures modify the contact interface morphology and facilitate lubricant guidance, while the circular micro-pits contribute to localized oil storage and debris sequestration. This functional integration reduces the recurrent re-entrainment of abrasive particles into the primary contact zone and alleviates the extent of local deformation and material transfer. When the textural parameters are favorably matched, the development of abrasive wear grooves and localized deformation can be effectively suppressed, thereby enhancing the overall tribological performance of the surface.

### 4.5. Discussion of Friction Reduction and Anti-Wear Mechanisms

By synthesizing the results of the friction coefficient (COF), wear behavior, and surface micro-morphology, it is evident that the biomimetic sinusoidal-circular composite micro-textures improved the tribological performance of 40Cr steel under oil-lubricated conditions. This improvement in friction reduction and wear resistance is primarily attributed to the combined effects of interfacial contact modulation, lubricant guidance and storage, and debris sequestration and transport.

The sinusoidal textures regulate the contact morphology of the friction interface and help reduce the real contact area. During sliding, these features facilitate the transport and redistribution of the lubricant along the texture orientation, thereby decreasing the probability of direct asperity-to-asperity contact. Meanwhile, the circular micro-pits act as localized oil reservoirs and provide space for debris entrapment. This function helps reduce the recurrent re-entrainment of abrasive particles into the primary contact zone and weakens secondary damage caused by abrasive particles. The combination of these two textural features therefore promotes a more stable lubrication condition while also alleviating local stress concentration.

When the geometric parameters are favorably matched, the composite micro-textures can simultaneously achieve lower COF values and milder surface damage. For instance, specimen L6 exhibited relatively better results in terms of steady-state average COF, relative wear severity, and post-test surface morphology, indicating that its parameter combination provided an effective synergistic effect in terms of lubricant guidance, oil storage, debris capture, and contact-state regulation. In contrast, when the parameter combinations were less favorable, the ability of the textures to maintain lubrication stability and suppress interfacial damage was weakened, resulting in higher COF values, more pronounced abrasive grooves, and more evident local deformation and material transfer.

It should be noted that the present tribological results were obtained under a comparative screening condition based on reciprocating ball-on-flat testing. Therefore, although the results are useful for evaluating the relative tribological performance of different texture parameter combinations, they do not directly represent the actual contact state of shafts or gears in service. Further investigation under more application-relevant contact conditions would be valuable in future work.

In summary, the sinusoidal textures regulate the contact interface and promote lubricant transport, while the circular micro-pits provide localized oil storage and debris sequestration. Their synergistic interaction improves the interfacial lubrication state, reduces local stress concentration, and suppresses the progression of abrasive wear together with localized deformation and material transfer. These findings indicate that the rational design of geometric parameter combinations for sinusoidal and circular textures is a key factor in improving the tribological performance of 40Cr steel under oil-lubricated conditions.

## 5. Conclusions

In this study, biomimetic composite micro-textures consisting of sinusoidal grooves and circular micro-pits were designed and fabricated based on the corrugated surface architecture of the Fimbria fimbriata shell. Their effects on the tribological performance of 40Cr steel under oil-lubricated conditions were systematically investigated. The main conclusions are as follows:

(1) A biomimetic composite micro-texture model combining sinusoidal and circular features was established based on the corrugation characteristics extracted from the *Fimbria fimbriata* shell. Through multi-region parameter measurement, statistical normalization, and orthogonal design, the parameter ranges of sinusoidal amplitude, wavelength, texture width, and circular dimple diameter were determined, providing the design basis for subsequent fabrication.

(2) Laser power had a marked influence on the cross-sectional forming quality of the sinusoidal textures. As the laser power increased, the groove depth generally increased, whereas the sidewall angle first increased and then decreased, and the edge transition width first decreased and then increased. Under the present experimental conditions, 4.50 W provided the most favorable overall cross-sectional morphology, with groove depth close to the target value, relatively steep sidewalls, and clear texture boundaries, and was therefore selected as the formal processing power for the sinusoidal textures.

(3) Under oil-lubricated reciprocating sliding conditions, all biomimetic sinusoidal-circular composite micro-textured specimens exhibited lower steady-state average COF values than the untextured specimen. Among them, specimen L6 showed the lowest steady-state average COF. Range analysis of the steady-state average COF values indicated that the influence of the factors followed the order of B > C > D > A, and the theoretical favorable parameter combination was A1 B3 C1 D1. These results suggest that rational texture parameter matching is beneficial for improving interfacial lubrication stability and reducing friction.

(4) White light interferometry results showed that the composite micro-textures generally improved the wear behavior of the 40Cr surface. Compared with the untextured specimen, most textured specimens exhibited shallower wear scars and lower relative wear severity. Using the relative displacement volume ratio (*K_w_*) as the normalized wear indicator, specimen L6 showed the lowest relative wear severity, indicating favorable antiwear performance under the present test conditions.

(5) Micro-morphological analysis before and after friction showed that abrasive wear was the dominant wear mode, accompanied by localized plastic deformation and material transfer features. Favorably matched composite textures could suppress the development of abrasive grooves and alleviate local surface damage. The friction reduction and antiwear mechanisms are mainly attributed to the synergistic effects of sinusoidal-groove-guided lubricant transport, circular-micro-pit-assisted oil storage and debris trapping, and the resulting regulation of the interfacial contact state. The present results were obtained under a comparative screening condition and provide experimental support for the further optimization and engineering application of biomimetic composite micro-textures on 40Cr steel surfaces.

## Figures and Tables

**Table 1:** Factors and levels for the Taguchi L9 design (adapted from Ref. [33]).

Factor	Parameter Name	Level 1	Level 2	Level 3
*A*	Sinusoidal amplitude (μm)	800	1000	1200
*B*	Sinusoidal wavelength (μm)	2000	2500	3000
*C*	Texture width (μm)	200	250	300
*D*	Circular dimple diameter (μm)	40	60	80

**Table 2:** L9 orthogonal array/parameter combinations (adapted from Ref. [33]).

Run ID	*A* (μm)	*B* (μm)	*C* (μm)	*D* (μm)
L1	800	2000	200	40
L2	800	2500	250	60
L3	800	3000	300	80
L4	1000	2000	250	80
L5	1000	2500	300	40
L6	1000	3000	200	60
L7	1200	2000	300	60
L8	1200	2500	200	80
L9	1200	3000	250	40

**Table 3:** Laser processing parameters for sinusoidal texture power optimization.

Laser Parameters	Values
Laser power (W)	3.75, 4.00, 4.25, 4.50, 4.75, 5.00
Repetition rate (kHz)	400
Hatch spacing (μm)	5
Number of scans	75
Beam quality (M^2^)	<1.3

**Table 4:** Parameters of the reciprocating friction and wear tests.

Experimental Parameter	Value
Experimental Apparatus	Bruker CETR UMT-2 (Bruker, Nanjing, China)
Normal Load (N)	150
Reciprocating Stroke (mm)	10
Frequency (Hz)	5.0
Average Linear Velocity (mm/s)	100

**Table 5:** Cross-sectional geometric parameters of sinusoidal textures under different laser powers.

Laser Power/W	Measured Depth *h* (μm)	Sidewall Horizontal Projection Δ*x* (μm)	Sidewall Angle *θ* (°)	Edge Transition Width *w_e_* (μm)
3.75	70	35	63	18
4.00	95	28	73	14
4.25	100	22	78	11
4.50	98	20	79	9
4.75	115	26	75	16
5.00	120	35	65	20

**Table 6:** Range analysis of steady-state average COF values.

Factor	Level 1	Level 2	Level 3	Range (R)	Optimal Level
*A*	0.1730	0.1754	0.1779	0.0050	*A*1
*B*	0.1823	0.1749	0.1690	0.0133	*B*3
*C*	0.1718	0.1759	0.1786	0.0068	*C*1
*D*	0.1726	0.1779	0.1758	0.0053	*D*1

**Table 7:** WLI volumetric parameters and relative displacement volume ratios for different specimens.

Run ID	Natural Volume (×10^6^ μm^3^)	Total Displaced Volume (×10^6^ μm^3^)	*K_w_*
UT	44.871	26.983	0.601
L1	43.328	27.303	0.630
L2	51.148	16.181	0.316
L3	25.895	6.990	0.270
L4	18.774	9.749	0.519
L5	27.897	14.038	0.503
L6	21.790	5.555	0.255
L7	67.278	31.835	0.473
L8	57.567	25.923	0.450
L9	45.960	22.355	0.486

## Data Availability

The original contributions presented in this study are included in the article. Further inquiries can be directed to the corresponding author.

## References

[1] Li Z., Li J., An B., Li R. (2024). The design method for surface texture of sliding friction pairs based on machine learning under mixed lubrication. Tribol. Int..

[2] Bowen L.K., Zuetell E., Long R., Rentschler M.E. (2025). Effects of Micropillar Array Strain on Sliding Friction at Soft Interfaces. ACS Appl. Eng. Mater..

[3] Wang P., Liang H., Jiang L., Qian L. (2023). Effect of nanoscale surface roughness on sliding friction and wear in mixed lubrication. Wear.

[4] Zhang T., Zhang F., Yin X., Han W., Zhang C., Chen H., Xiong B., Yang K., Hao Y. (2022). Important explorations of the sliding tribological performances of micro/nano-structural interfaces: Cross-shaped micro-concave and the nanoNb2AlC-Sn. Eng. Fail. Anal..

[5] Xie X., Zeng Z., Luo J., Xu J. (2021). Friction and wear characteristics of linear contact sliding friction pairs under oil-air lubrication. J. Braz. Soc. Mech. Sci. Eng..

[6] Xue L., Yan Z., Jiang Y., Sun T. (2024). Influences of Sharkskin Texture on Lubrication Performance of Elastic Bearing Friction Pairs. Tribol. Lett..

[7] Yang H., Yang X., Cong J., Sun J., Shao S., Hou Q., Zhang Y. (2023). Wear Behavior of Microgroove Texture of Cemented Carbide Tool Prepared by Laser Surface Texture. SSRN Electron. J..

[8] Su B., Ye J., Zou X., Huang L., Wang X. (2023). Effect of bionic hexagonal texture on squeezing films inside soft contacts with high adhesion and high friction. Soft Matter.

[9] Wang Y., Peng W., Tong H., Sun Y., Liu Z., Wu F. (2024). A biomimetic micro-texture based on shark surface for tool wear reduction and wettability change. J. Manuf. Process..

[10] Yin H., Yang J., Gu Q. (2024). Numerical study on the hydrodynamic lubrication performance improvement of bio-inspired peregrine falcon wing-shaped micro-texture. Tribol. Int..

[11] Li J., Zhao Y., Zhang X., Su J., Tan C., Yang J., Song X., Song W., Guo G. (2024). Influence of bionic texture on the mechanical properties of 6061Al/CFRTP laser joints. Thin-Walled Struct..

[12] Gao H., Shi X., Xue Y., Zhang K., Huang Q., Wu C. (2023). Optimization of Bionic Textured Parameters to Improve the Tribological Performance of TC4-Based Self-Lubricating Composite Using Response Surface Methodology. J. Mater. Eng. Perform..

[13] Kasman E., Ozan S. (2026). Marine Shell-Inspired Laser Surface Texturing: Characterizing the Surface Properties of Co-Based Alloy. J. Bionic Eng..

[14] Zhao J., Xue X., Yan Y., Sedao X. (2025). Experiment and simulation of femtosecond laser processing of peek materials: One-step laser optimization of friction performance and wettability. Opt. Laser Technol..

[15] Paula K.T.D., Lin H.I., Yang F., Vollet-Filho J.D., Gu T., Hu J., Mendonça C.R. (2024). Femtosecond laser processing of amorphous silicon films. J. Manuf. Process..

[16] Guo Y., Zhao H. (2024). Femtosecond laser processed superhydrophobic surface. J. Manuf. Process..

[17] Zhang W., Zhang P., Yan H., Li R., Shi H., Wu D., Sun T., Luo Z., Tian Y. (2023). Research status of femtosecond lasers and nanosecond lasers processing on bulk metallic glasses (BMGs). Opt. Laser Technol..

[18] Chen B., Chai N., Zhang J., Ren L., Wang W., Zhang F., Wang X., Fu Z. (2023). Wetting tuning of Al/B4C interface via femtosecond laser irradiation. Appl. Surf. Sci..

[19] Tan J.W., Wang G., Zhao G.X., Hou Y.C., Sun D.R., Song Y.F., Dong L.Y., Zhao H., Wang Y. (2022). Femtosecond laser hybrid processing strategy of transparent hard and brittle materials. Front. Chem..

[20] Zuo P., Zhu D., Li F., Tian H., Han W., Liu T., Yu K., Zhou J., He X. (2025). Fabrication of Superhydrophobic Micro/Nanostructures of Titanium Alloy by Femtosecond Laser. ACS Omega.

[21] Fan Z., He S., Yi L., Pei Z., Shen P., Ou W., Mei X. (2026). Femtosecond laser preparation and tensile behavior of taper/gully-free FCHs. Int. J. Mech. Sci..

[22] Wang L., Liu Y.A., Duan J., Bao Y. (2024). Performance analysis of columnar convex-concave compound micro-texture thrust bearing. Ind. Lubr. Tribol..

[23] Chen D., Cui Y., Sun K., Fan J., Cheng K. (2024). Coupling effect of partial composite texture and thermal effect on the performance of hydrostatic bearing. Lubr. Sci..

[24] Liang Y., Zhang F., Xing H., Wang C., Chen N., Wang W., Zhang Z. (2025). Lubrication and wear characteristics of bionic composite texture on friction pair under seawater condition. Surf. Topogr. Metrol. Prop..

[25] Deng L., Su J., Jin Z. (2024). Effect of composite textured rough surfaces on the lubrication performance of cylindrical roller bearings. Ind. Lubr. Tribol..

[26] Chen D., Li Y., Cao X., Wu T., Zhang H., Qiao Z., Niu C., Wang Y., Wang S., Ling C. (2023). Evaluating characteristics of particles surface micro-texture of granular materials based on the spectral analysis method. Int. J. Pavement Eng..

[27] McCray A.R., Cote T., Li Y., Petford-Long A.K., Phatak C. (2021). Understanding Complex Magnetic Spin Textures with Simulation-Assisted Lorentz Transmission Electron Microscopy. Phys. Rev. Appl..

[28] Mao Y., Zhang Y., Zheng J., Li L., Huang Y., Shi S., Wang L., Pei J., Li Z. (2024). A Study on Micro-Pit Texture Parameter Optimization and Its Tribological Properties. Machines.

[29] Yuan Q., Wang Z., Zhang Y., Ye J., Huang Y., Huang A. (2020). Effect of Warm Rolling Temperature on the Microstructure and Texture of Microcarbon Dual-Phase (DP) Steel. Met. Open Access Metall. J..

[30] Wang L., Du R.F., Zhang X.F., Jia C.Y., Cai A.T. (2025). Cracking Failure Analysis of 40Cr Drive Shaft. J. Fail. Anal. Prev..

[31] Li C., Deng S., Sun H., Han X. (2024). Optimization of laser irradiation process parameters for 40Cr steel based on back propagation neural network and genetic algorithm. Proc. Inst. Mech. Eng. Part L J. Mater. Des. Appl..

[32] Yao Y., Xiu S., Sun C., Kong X., Hong Y. (2021). Investigation on grinding-induced dynamic recrystallization behavior of 40Cr alloy steel. J. Alloys Compd. Interdiscip. J. Mater. Sci. Solid-State Chem. Phys..

[33] Chen Y., Xu P., Yu Y., Shen J. (2025). Optimization of Laser-Induced Composite Micro-Textures on PEEK/CF Composites and Their Wetting–Friction Behaviors. Lubricants.

